# Anisotropic Gold Nanoparticle-Cell Interactions Mediated by Collagen

**DOI:** 10.3390/ma12071131

**Published:** 2019-04-06

**Authors:** Oana T. Marișca, Nicolae Leopold

**Affiliations:** 1 Faculty of Physics, Babeș-Bolyai University, 400084 Cluj-Napoca, Romania; oanamarisca@gmail.com; 2 IMOGEN Research Institute, County Clinical Emergency Hospital, 400012 Cluj-Napoca, Romania

**Keywords:** gold nanoparticle, collagen, cellular viability, cellular internalization, SERS

## Abstract

Gold nanoparticles (AuNPs) are the groundwork of a large variety of applications in the biomedical field. Further development and a better understanding of this versatile platform will lead to an expansion of potential applications. In this study, we propose a facile synthesis of AuNPs using hydrogen peroxide as a reducing agent and collagen as a stabilizing agent. Our synthetic approach results in “raspberry”-like AuNPs with a mean diameter of 60 nm, as revealed by electron microscopy. The optical properties of the AuNPs were assessed by UV-Vis and surface-enhanced Raman scattering (SERS), and their stability and in vitro cytotoxicity were evaluated as well. HeLa cell viability values were only modestly affected compared to control, with the highest concentration tested displaying a 20% decrease in cellular viability. The dose-dependent cellular internalization in the 20–60 nM range indicate the highest internalization rate at 60 nM and uptake values as high as 35%. This result correlated well with the viability results. These type of anisotropic AuNPs are proposed for biomedical applications such as hyperthermia, contrast agents or imaging. Therefore, our findings offer a platform for potential biological applications such as sensing and imaging, due to their unique physico-chemical features.

## 1. Introduction

The development of new nanomaterials is an important pursuit of the scientific community due to the need to design multifunctional, and biocompatible nano-systems. In particular, the interaction of functional nanomaterials with living cells is of interest for the development of new therapeutic agents [1]. A specific area of focus is the investigation and optimization of gold nanoparticles (AuNPs), which were found to be versatile for further functionalization due to their physico-chemical properties, a facile synthesis process, and the ease of controlling their size and shape [1,2].

There are a large variety of synthesis methods for AuNPs that have reported so far. They have been categorized as chemical methods, seed mediated grown methods and green methods [3,4]. Seed mediated grown methods allow for a better size and shape control of the particle formed using very small gold templates and a suitable growth environment. However, this method falls short on ease of execution and the numerous steps involved. The most common method for AuNPs is the chemical one. Among the chemical synthesis category, the two most widely known and used methods are the Turchevich-Frens and Brust-Schriffin methods. The solvated gold salt is reduced in the presence of sodium citrate [5,6] or sodium borohydride [7], leading to spherical AuNPs formation. These methods have a lower degree of control, being mostly dependent on the gold to citrate/borohydride molar ratio. Some drawbacks of these two synthesis methods are reproducibility, difficult steps (boiling, phase separation), and stability of the particles in case of reduction in the presence of borohydride. There are several improved variations of the chemical synthesis reported that mitigate these disadvantages [8,9]. More recently, green synthesis methods were developed to improve *in vitro*/*in vivo* compatibility of AuNPs. These methods use bacteria, fungi, plant extracts proteins and amino acids together with outside energy sources like light, ultrasound and microwave. Even though the green methods yield less toxic nanoparticles there is still a need for more optimization of AuNPs characteristics control and scalability. 

When dealing with colloidal systems, their stability is the main concern [10]. Once the nanoparticles are formed, the next step in the protocol is their stabilization, regardless of the environment in which they are suspended [11]. An adequate choice would be a polymer or a protein that prevents nanoparticles from aggregating by adsorbing at their surface. This is also well described in the recent idea of protein corona formation when nanoparticles are exposed to biological fluids [12]. Moreover, by having a protein as a layer on top of the nanoparticles, the options for further applications expands [13]. Proteins are a good source of different functional groups that can serve as binding sites for other molecules such as dyes, antibodies or drugs [14]. This way, with reduced procedural steps, a multifunctional nanomaterial can be designed.

In the present approach, a naturally occurring protein, collagen, was chosen as a versatile coating. It combines all mandatory characteristics for biological applications, namely, biocompatibility, accessibility and safety. After synthesis, the next step is to assess the toxic effect of the newly developed AuNPs in *in vitro* systems. It has been reported that the physical characteristics of the nanoparticles influence their cellular interaction [15]. Among these, the size, shape and surface net charge have a great contribution to the possible outcomes in cell-nanoparticle interplay.

In this study, we report a new, single-step synthesis method of generating AuNPs by using collagen as a stabilizing agent. When mixing collagen with hydrogen peroxide, the reduction process occurs at room temperature leading to anisotropic, 60 nm diameter, raspberry-like shaped AuNPs, also known as clg-AuNPs. This novel synthetic procedure is highly reproducible, employs mild conditions, leads to stable nanoparticles that do not aggregate and additionally can be potentially further optimized via functionalization of the collagen coating on its surface. The obtained AuNPs were characterized by ultraviolet-visible (UV-Vis) absorption spectroscopy, transmission electron microscopy (TEM) and dynamic light scattering (DLS). The biological effects on HeLa cells were assessed by MTT assay and by determining the rate of internalization using inductive coupled plasma-mass spectrometry (ICP-MS).

## 2. Materials and Methods 

### 2.1. Gold Nanoparticle Synthesis

For the AuNPs synthesis, a stock solution of gold salt was prepared by solving 1 g of hydrogen tetrachloroaurate(III) hydrate metal basis (Alfa Aesar, Ward Hill, MA, USA; 99.9% purity) in 50 mL ultrapure water. In parallel, a stock solution of collagen was prepared by mixing 10mL ultrapure water with 0.02 g collagen type I from bovine Achilles tendon (Sigma-Aldrich, St. Louis, MI, USA) in the presence of 500 µL, 37% hydrochloric acid (Sigma Aldrich). 1% Sodium hydroxide (Fluka, Munich, Germany) and 3% hydrogen peroxide solution (AppliChem, Darmstadt, Germany) were also prepared alongside these stock solutions. Further, the mixing steps will be detailed below. All solutions were prepared in MilliQ ultrapure water (Direct-Q 3 UV, Millipore, Burlington, MA, USA) with a resistivity higher than 18 MΩ.

It must be mentioned that the reduction of the gold ions could not be achieved using a simple collagen aqueous solution. Therefore, prior to the addition of the gold salt, hydrogen peroxide was added to the protein aqueous solution, as described below.

**Figure 1:**
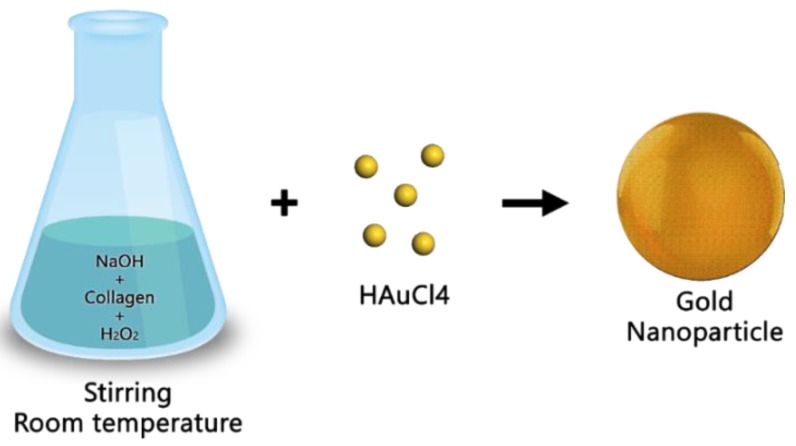
A schematic overview of the clg-AuNPs synthesis process.

First, 500 µL of the prepared stock collagen aqueous solution was mixed with 500 µL of 3% hydrogen peroxide solution in a tube. Separately, to a 600 µL aliquot of the gold salt stock solution was added 1 mL of 1% sodium hydroxide solution. These two final solutions were then both added to 90 mL ultrapure water in an Erlenmeyer flask by stirring at room temperature. After fews, the solution turned blue and had a pH value of 9. Figure 1 provides a schematic overview of the above described synthesis procedure.

### 2.2. Analysis

UV-Vis spectra of the prepared gold colloids were recorded on a Jasco V-630 UV-Vis spectrophotometer (Pfungstadt, Germany), by using quartz cuvettes with an optical pathlength of 1 cm.

The surface-enhanced Raman scattering (SERS) spectra were recorded at a spectral resolution of 8 cm^−1^ using an Advantage 200A Raman spectrometer (DeltaNu, Laramie, WY, USA), equipped with a 5 mW, HeNe laser emitting at 633 nm. 

Size and morphology of the AuNPs were determined using transmission electron microscopy (TEM) imaging, performed on a JEOL model JEM 1010 microscope (Tokyo, Japan). High resolution TEM micrographs of the AuNPs were recorded on a PHILIPS CM 20 microscope (Amsterdam, The Netherlands) operated at 200 kV. The diameters were calculated from the TEM micrographs using the ImageJ 1.45s software (Wayne Rasband, National Institutes of Health (NIH), Bethesda, MD, USA).

The hydrodynamic diameter distribution and zeta potential values were obtained using a Zetasizer Nano ZS (Malvern Instruments, Malvern, UK). The dynamic light scattering (DLS) measurements were performed in a disposable sizing cuvette under a 173° backscattering operating mode.

The inductively coupled plasma mass spectrometry (ICP-MS) measurements were realized using the ICP-MS 7700 model form Agilent (Santa Clara, CA, USA). The samples were introduced into the ICP-MS using a Peltier Cooled Cyclonic Spray Chamber for Agilent ICP-MS 7700 series as a fine mist to ensure a perfect distribution of a sample solution inside the plasma and to control the sample quantity which is introduced at the same time for perfect ionization. After ionization, the sample was introduced into the quadrupole through an Omega lens system which consists of charged conic lenses. Inside the quadrupole, the ions are forced into a horizontal spiral depending on their charge to mass ratio and are either directly separated from the measured elements, or lose so much kinetic energy that they are not able to pass the final kinetic barrier and the second part of charged lenses before the detector. All reported values are means of 6 individual measurements. Between samples the spray chamber and the tube system were cleaned using MilliQ water for 20 s, then 2% HCl for 30 s and again MilliQ water for at least 40 s at a flow of 0.5 mL per second.

### 2.3. In Vitro Experimental Methods

Prior to all biological investigations, the nanoparticles were characterized using DLS measurements. DLS provides information about uniformity of the size distribution of colloidal samples and can offer a view on the cluster formation when AuNPs are dispersed in cellular culture medium. Also, assessment of the Zeta potential value, in other words the net charge at the surface of nanoparticles, is indicative of the colloidal stability of nanoparticles and can predict a favorable interaction of the AuNPs with the cellular membrane. 

For *in vitro* experiments, cervical carcinoma (HeLa) cells were cultured in Dulbecco’s Modified Eagle’s culture medium (DMEM, Sigma Aldrich) supplemented with 2 mM glutamine, 10% fetal bovine serum (FBS) and 100 U/mL penicillin/streptomycin. The cells were grown at 37 °C in a humidified atmosphere containing 5% CO_2_. Cells were seeded on 96-well plates with a 5000 cells/well density, 24 h before experiments.

Toxicity induced by AuNPs was assessed by the MTT assay. HeLa cells were incubated for 4 h with different concentrations of AuNPs. After that, the medium with the excess particles was removed and the cells were cultured for additional 20 h. An MTT assay (Roche, Basel, Switzerland) was then performed according to the manufacturer´s instructions.

For uptake experiments, the cells were incubated with different concentrations of AuNPs for 4 h. The supernatants were collected and frozen for further analysis. Fresh medium was added to the cells followed by an additional 20 h incubation. Then the medium was removed, and the cells were lysed with a lysis buffer. After 30 min the samples were collected and stored at −20 °C. Next, acid digestion of samples was performed. For this, a mixture of 150 µL, 37% HCl and 50 µL, 65% HNO_3_ was prepared. After 5 min, this mixture was added to each sample. This was followed by the addition of 2.7 mL, 2% HCl. This treatment ensures sample decomposition to near atomic size and allows more accurate ICP-MS measurements.

## 3. Results and Discussion

**Figure 2:**
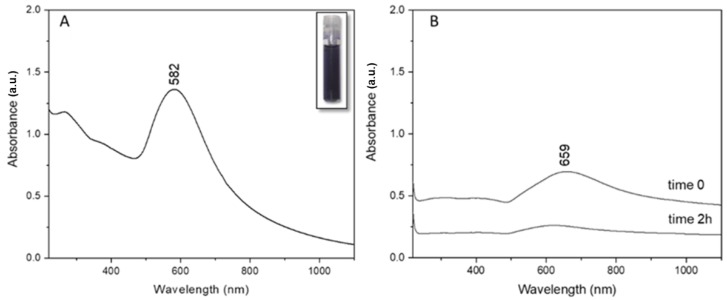
(**A**) UV-Vis spectrum of clg-AuNPs colloid (the inset shows a photo of the colloid in a vial); (**B**) The UV-vis spectra of mixture of gold salt and H_2_O_2_ without using any collagen, recorded right after synthesis (time 0) and after 2 h.

Post synthesis, the UV-Vis absorption profile of clg-AuNPs was registered (Figure 2A). The clg-AuNPs have a maximum of absorption at 582 nm, which correlates directly with the colloid’s deep blue color, as shown in the inset of Figure 2A. The plasmon band is significantly shifted towards higher wavelengths compared to the absorption maximum of common spherical gold colloids at around 520 nm [16], suggesting that the clg-AuNPs might have a bigger size and anisotropic shape.

Since there have been reports in the literature about methods in which hydrogen peroxide is used as a reducing agent for AuNPs [17,18], it was considered necessary to demonstrate the stabilizing role of collagen, critical for the formation of our described nanoparticles. Therefore, as a control measurement, we reproduced the exact synthesis steps mentioned in the Methods section but without the addition of collagen. The plasmon band of the obtained solutions was measured via UV-Vis absorption, with spectra represented in Figure 2B. When the gold salt was mixed with the hydrogen peroxide solution, a plasmon band rises around 660 nm. This indicates that gold salt is reduced and some type of AuNPs might be formed. The colloidal solution obtained had a greenish color with an orange haze. According to the literature, gold nanoparticles that absorb at such high wavelengths have a high degree of anisotropy, like gold nanorods [19]. However, it was noticed that the obtained colloidal mixture started to become transparent over time. Therefore, the absorption profile was re-obtained at 2 h post-synthesis and it was observed that the initial plasmon band at 659 nm decreased considerably in intensity, suggesting aggregation of these nanoparticles. 

Thus, in this specific synthesis method, collagen proves essential for clg-AuNPs formation. The UV-Vis absorption profile of clg-AuNPs nanoparticles shows a maximum of absorption at 582 nm; however, the plasmonic band is very broad, and no physical characteristics about size or shape could be concluded based only on the plasmonic band position. 

**Figure 3:**
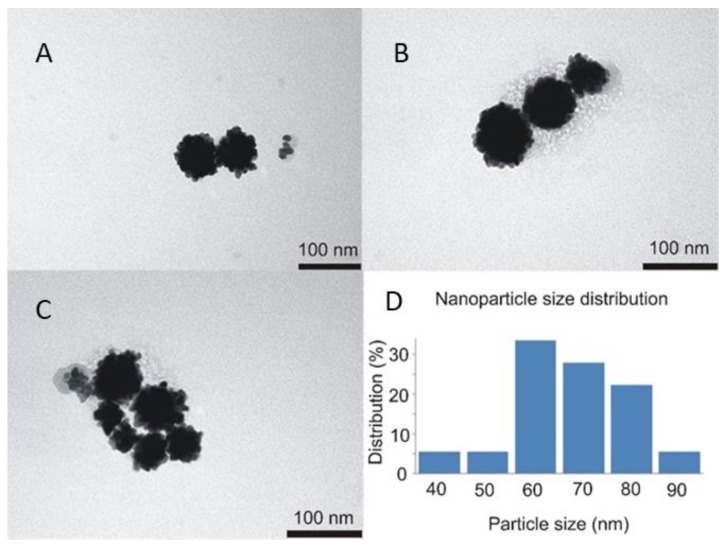
Representative TEM images of clg-AuNPs nanoparticles (**A**–**C**) and their size distribution (**D**).

The next step in clg-AuNPs characterization was imaging their structure and assessing their size via TEM imaging and DLS measurements. The TEM images of clg-AuNPs (Figure 3) show that their shape is anisotropic, similar to a raspberry [18]. According to the nanoparticle distribution, calculated with ImageJ software from TEM images, their diameter distribution peaks around 60 nm, with 80% of the population being in the 60 to 80 nm range (Figure 3D).

**Figure 4:**
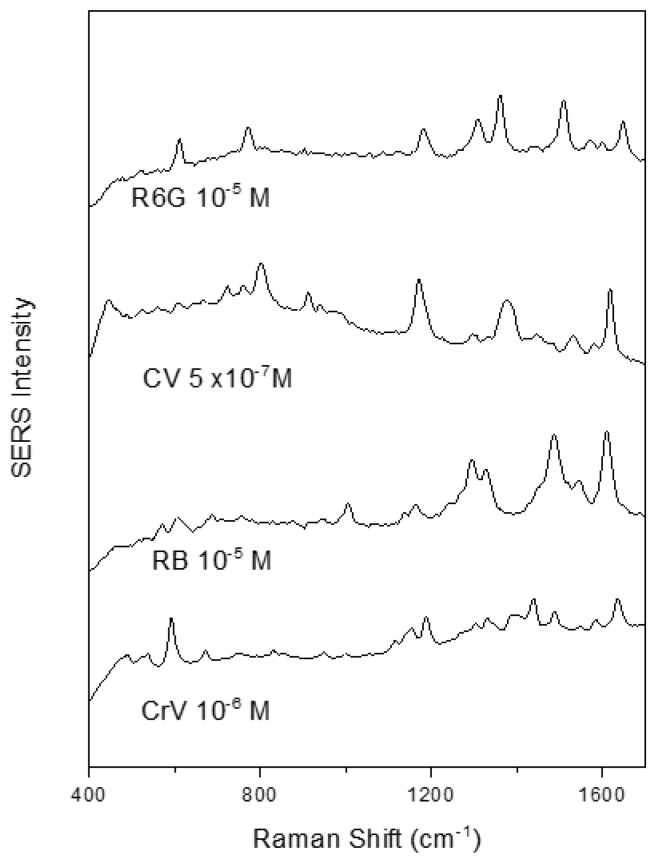
SERS spectra of rhodamine 6 G (R6G), crystal violet (CV), rose bengal (RB) and cresyl violet (CrV) having as substrate the clg-AuNPs.

Furthermore, the Raman scattering enhancement activity of these nanoparticles was found to be comparable with that of conventional colloids. Figure 4 shows SERS spectra of several cationic dyes with a high signal to noise ratio when using the clg-AuNPs as SERS substrate and the test analytes at 10^−5^ M or lower concentrations.

To note, high quality SERS spectra could be recorded even in the 10^−7^ M concentration range, using crystal violet as test analyte. The SERS spectral shape of the here recorded analytes is in concordance with the spectra recorded using gold colloids synthesized by other reducing agents such as polyethylene glycol [20] or glucose [21], indicating that the raspberry shape of the nanoparticles and the collagen surfactant do not influence the adsorption geometry of the test analytes.

No noticeable time delay between analyte addition and SERS spectra appearance was observed, suggesting that these analytes could easily penetrate the collagen layer and adsorb to the gold surface. It must be mentioned that the clg-AuNPs colloid shows no intrinsic SERS signal, the only bands are due to water Raman scattering.

According to the size distribution measured by DLS, the mean hydrodynamic diameter was around 70 nm and the polydispersity index value of 0.203 suggests a broad size distribution of these AuNPs population. The Zeta potential value was found to be −47 mV, which indicates high colloidal stability (data shown in supplementary information Figure S1).

The nanoparticles´ size and shape are known to influence their interaction with cells [1]. In particular, anisotropic nanoparticles were found to be less preferred for uptake as compared to spherical nanoparticles [22]. Also, AuNP´s coating was shown to play an important role in AuNPs uptake and toxicity [23]. Therefore, in the following set of experiments we studied the uptake and toxicity of the clg-AuNPs. In the *in vitro* experiments, the cells were exposed to the AuNPs in cell culture medium. This medium contains high salt concentrations, antibiotics and a cocktail of proteins. It is well known that proteins or salts present in cell culture media can interact with AuNPs, leading to changes in their size by forming aggregates and becoming unstable [24,25].

Therefore, DLS measurements were performed on clg-AuNPs dispersed in cell culture DMEM medium without Phenyl Red, which was supplemented or depleted of FBS. The concentration of clg-AuNPs was 100 nM and the ratio of DMEM to clg-AuNPs was 9:1 by volume. The measurements were performed three times, and the average of these measurements is reported in Table 1. The particle distribution profile is presented in supplementary information Figure S2. 

The clg-AuNPs were stable in protein-containing medium. Under these conditions, the hydrodynamic diameter did not increase dramatically over time, suggesting that the presence of other proteins does not affect these particles. While the mean diameter slightly increased, it is believed that this is due to proteins from the serum forming a protein “corona” around the particles [26,27].

However, when the clg-AuNPs were exposed to DMEM without FBS, their mean size increased, suggesting that these particles form aggregates. We believe that the high salt concentrations affect the stability of these particles. Literature reports suggest that some AuNPs are unstable at salt concentration higher than 100 mM [28,29], and, for instance, the sodium chloride concentration in DMEM is around 110 mM. Nevertheless, these findings do not affect any potential *in vivo* use of the clg-AuNPs. For example, these nanoparticles would be a good candidate for intravenous administration, since they are so stable in presence of serum-containing media, therefore the natural biological environment present in blood would not affect their stability and trafficking.

The beforehand performed stability tests suggest that clg-AuNPs are appropriate to use for *in vitro* testing. Therefore, HeLa cells were exposed to clg-AuNPs for toxicity and uptake experiments.

**Figure 5:**
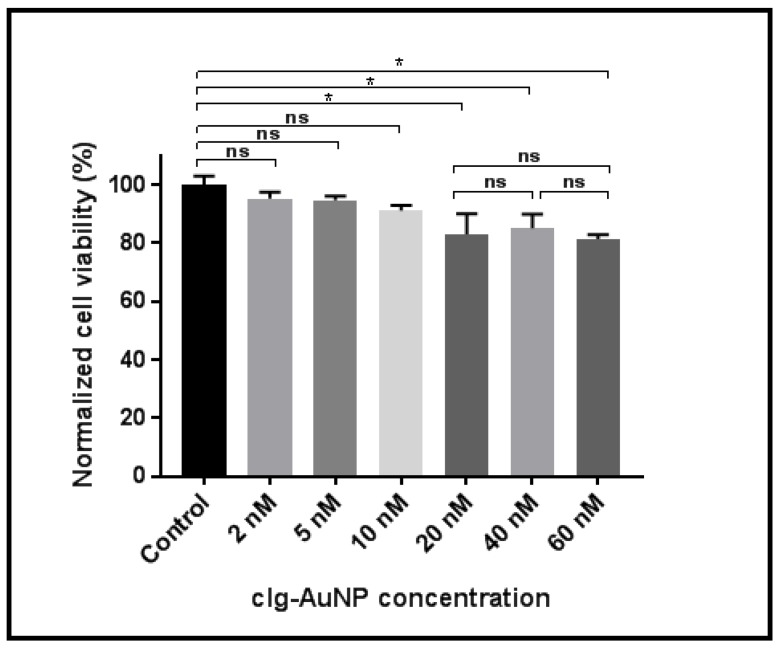
HeLa cellular viability at different clg-AuNPs concentration. Cellular viability was compared to untreated, control sample, and additionally in between groups, using ANOVA with multiple hypothesis testing (* *p* < 0.05).

Figure 5 shows HeLa cellular viability as a function of clg-AuNPs concentration. HeLa cells were incubated with nanoparticles at concentrations ranging from 5 nM to 60 nM. The groups were statistically analyzed using one-way analysis of variance (ANOVA), employing Tukey correction for multiple comparisons between groups using statistical hypothesis testing. The reported p values are multiplicity adjusted to account for multiple comparisons, with the family-wise significance and confidence level set at 0.05 (95% confidence interval).

Analyses revealed a statistically significant decrease in cellular viability starting with the 20 nM concentration of nanoparticles. However, this effect did not exceed a 20% decrease in cellular viability, and furthermore, there was no statistically significant difference between the 20 nM concentration and the highest tested concentration (60 nM). These data suggest that the nanoparticles have a modest impact on cellular viability *in vitro*. 

**Figure 6:**
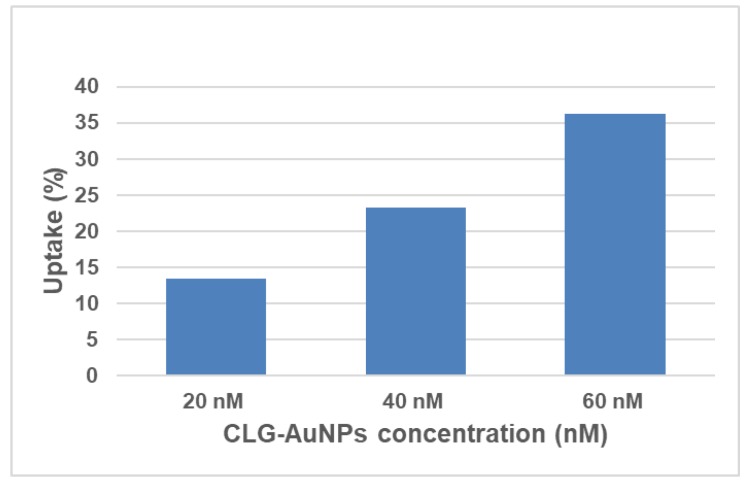
Internalization of clg-AuNPs in HeLa cells.

Since the clg-AuNPs nanoparticles exhibit low cytotoxicity, uptake studies were performed in order to further understand their effects on cells. Figure 6 shows the percentage of internalized gold in the cells from the total concentration of gold that was administered to the HeLa cells. The highest uptake of clg-AuNPs is around 35% for the highest concentration tested (60 nM).

The uptake levels were proportional with the concentration the cells were exposed at. However, the internalization percentages in HeLa cells are moderately low for these clg-AuNPs. This fact is consistent with results from other reports, which indicate that anisotropic AuNPs preponderantly display a lower degree of uptake compared with spherical AuNPs, which are more efficiently internalized [30,31].

Our findings indicate that these anisotropic gold nanoparticles might not have an advantage over spherical gold nanoparticles for delivery purposes; however, their value resides in their unique optical properties. Applications for anisotropic gold nanoparticles are also described in other publications [32,33]. Possible biomedical applications include diagnostic testing, since these clg-AuNPs have a unique surface plasmon band position which can be further exploited. Also, these nanoparticles are good SERS substrates, which could serve as platforms for detecting trace amounts of antibodies, hormones or drugs, with potential applications in clinical medicine. More recently, AuNPs have been used as imaging contrast agents [34]; our particles could also be used for this purpose, since they are easily synthesized and are very stable. Lastly, clg-AuNPs can be used for hyperthermia in cancer therapy [35]: being metallic nanoparticles, they conduct heat well; plus, these raspberry-shaped anisotropic nanoparticles display a plasmon band position at 582 nm, closer to the Near Infra-Red region, and their efficiency in converting light to heat should be higher compared to other gold nanoparticles. 

## 4. Conclusions

We report a simple, one-step preparation method at room temperature to obtain a new type of AuNPs. Our method uses hydrogen peroxide as reducing agent and collagen as stabilizing agent. The obtained nanoparticles have an intriguing anisotropic “raspberry” shape with a mean diameter of 60 nm, and they are stable in culture media. The viability results indicate low *in vitro* toxicity, with uptake values as high as 35%, displaying a minimal effect on cellular viability. 

This novel synthesis procedure is highly reproducible, employs mild room temperature conditions and leads to stable nanoparticles that do not aggregate in cell culture media. Together, these findings suggest that these novel anisotropic collagen-coated gold nanoparticles present a viable platform for potential biological applications such as diagnostic testing, sensing and imaging, due to their unique and favorable physico-chemical features.

## Figures and Tables

**Table 1:** Hydrodynamic diameter of clg-AuNPs dispersed in DMEM w/ and w/o FBS at three different time points. Mean value and standard deviation of three measurements per condition are reported.

Cell Culture Medium	0 h	2 h	4 h
DMEM w/ FBS	83 ± 3.01 nm	81 ± 2.82 nm	95 ± 2.96 nm
DMEM w/o FBS	114 ± 3.66 nm	468 ± 4.9 nm	763 ± 4.79 nm

## Supplementary Materials

The following are available online at https://www.mdpi.com/1996-1944/12/7/1131/s1, Figure S1. Size distribution of clg-AuNPs measured by dynamic light scattering (DLS) (A). Zeta potential distribution of clg-AuNPs (B). Figure S2. DLS measurements showing clg-AuNPs stability in cell culture medium without or with serum.

## References

[1] Giljohann D.A., Seferos D.S., Daniel W.L., Massich M.D., Patel P.C., Mirkin C.A. (2010). Gold Nanoparticles for Biology and Medicine. Angew. Chem. Int. Ed..

[2] Khlebtsov N.G., Dykman L.A. (2010). Optical properties and biomedical applications of plasmonic nanoparticles. J. Quant. Spectrosc. Radiat. Transf..

[3] Herizchi R., Abbasi E., Milani M., Akbarzadeh A. (2014). Current methods for synthesis of gold nanoparticles. Artif. Cells Nanomed. Biotechnol..

[4] Qin L., Zeng G., Lai C., Huang D., Xu P., Zhang C., Cheng M., Liu X., Liu S., Li B. (2018). “Gold rush” in modern science: Fabrication strategies and typical advanced applications of gold nanoparticles in sensing. Co-ord. Chem. Rev..

[5] Turkevich J., Stevenson P.C., Hillier J. (1951). A study of the nucleation and growth processes in the synthesis of colloidal gold. Discuss. Soc..

[6] Frens G. (1973). Controlled Nucleation for the Regulation of the Particle Size in Monodisperse Gold Suspensions. Nat. Phys. Sci..

[7] Brust M., Walker M., Bethell D., Schiffrin D.J., Whyman R. (1994). Synthesis of thiol-derivatised gold nanoparticles in a two-phase Liquid-Liquid system. J. Chem. Soc. Chem. Commun..

[8] Anderson M.J., Torres-Chavolla E., Castro B.A., Alocilja E.C. (2010). One step alkaline synthesis of biocompatible gold nanoparticles using dextrin as capping agent. J. Nanoparticle Res..

[9] Tódor I.S., Szabó L., Marişca O.T., Chiş V., Leopold N. (2014). Gold nanoparticle assemblies of controllable size obtained by hydroxylamine reduction at room temperature. J. Nanoparticle Res..

[10] Krumpfer J.W., Schuster T., Klapper M., Müllen K. (2013). Make it nano-Keep it nano. Nano Today.

[11] Hühn D., Kantner K., Geidel C., Brandholt S., De Cock I., Soenen S.J.H., Rivera_Gil P., Montenegro J.-M., Braeckmans K., Müllen K. (2013). Polymer-Coated Nanoparticles Interacting with Proteins and Cells: Focusing on the Sign of the Net Charge. ACS Nano.

[12] Del Pino P., Pelaz B., Zhang Q., Maffre P., Nienhaus G.U., Parak W.J. (2014). Protein corona formation around nanoparticles – from the past to the future. Mater. Horizons.

[13] Sanpui P., Paul A., Chattopadhyay A. (2015). Theranostic potential of gold nanoparticle-protein agglomerates. Nanoscale.

[14] Slocik J.M., Naik R.R. (2017). Sequenced defined biomolecules for nanomaterial synthesis, functionalization, and assembly. Curr. Opin. Biotechnol..

[15] Marisca O.T., Kantner K., Pfeiffer C., Zhang Q., Pelaz B., Leopold N., Parak W.J., Rejman J., Selvan S.T. (2015). Comparison of the in Vitro Uptake and Toxicity of Collagen- and Synthetic Polymer-Coated Gold Nanoparticles. Nanomaterials.

[16] Haiss W., Thanh N.T.K., Aveyard J., Fernig D.G., Hanson J. (2007). Determination of Size and Concentration of Gold Nanoparticles from UV−Vis Spectra. Anal. Chem..

[17] Li Q., Lu B., Zhang L., Lu C. (2012). Synthesis and stability evaluation of size-controlled gold nanoparticles via nonionic fluorosurfactant-assisted hydrogen peroxide reduction. J. Mater. Chem..

[18] Raula M., Maity D., Rashid M.H., Mandal T.K. (2012). In situ formation of chiral core–shell nanostructures with raspberry-like gold cores and dense organic shells using catechin and their catalytic application. J. Mater. Chem..

[19] Rayavarapu R.G., Petersen W., Ungureanu C., Post J.N., Van Leeuwen T.G., Manohar S. (2007). Synthesis and Bioconjugation of Gold Nanoparticles as Potential Molecular Probes for Light-Based Imaging Techniques. Int. J. Biomed..

[20] Leopold N., Chiş V., Mircescu N.E., Marişca O.T., Buja O.M., Leopold L.F., Socaciu C., Braicu C., Irimie A., Berindan-Neagoe I. (2013). One step synthesis of SERS active colloidal gold nanoparticles by reduction with polyethylene glycol. Colloids Surf. A Physicochem. Eng. Asp..

[21] Boitor R.A., Tódor I.S., Leopold L.F., Leopold N. (2015). Room Temperature Synthesis of Highly Monodisperse and Sers-Active Glucose-Reduced Gold Nanoparticles. J. Appl. Spectrosc..

[22] Florez L., Herrmann C., Cramer J.M., Hauser C.P., Koynov K., Landfester K., Crespy D., Mailänder V. (2012). How Shape Influences Uptake: Interactions of Anisotropic Polymer Nanoparticles and Human Mesenchymal Stem Cells. Small.

[23] Murphy C.J., Gole A.M., Stone J.W., Sisco P.N., Alkilany A.M., Goldsmith E.C., Baxter S.C. (2008). Gold Nanoparticles in Biology: Beyond Toxicity to Cellular Imaging. Accounts Chem. Res..

[24] Pyshnaya I.A., Razum K.V., Poletaeva J.E., Pyshnyi D.V., Zenkova M.A., Ryabchikova E.I. (2014). Comparison of Behaviour in Different Liquids and in Cells of Gold Nanorods and Spherical Nanoparticles Modified by Linear Polyethyleneimine and Bovine Serum Albumin. BioMed Res. Int..

[25] Chanana M., Rivera_Gil P., Correa-Duarte M.A., Liz-Marzán L.M., Parak W.J. (2013). Physicochemical Properties of Protein-Coated Gold Nanoparticles in Biological Fluids and Cells before and after Proteolytic Digestion. Angew. Chem. Int. Ed..

[26] Gebauer J.S., Malissek M., Knauer S.K., Treuel L., Simon S., Maskos M., Stauber R.H., Peukert W. (2012). Impact of the Nanoparticle–Protein Corona on Colloidal Stability and Protein Structure. Langmuir.

[27] Casals E., Pfaller T., Duschl A., Oostingh G.J., Puntes V. (2010). Time Evolution of the Nanoparticle Protein Corona. ACS Nano.

[28] Johnston B.D., Kreyling W.G., Pfeiffer C., Schäffler M., Sarioglu H., Ristig S., Hirn S., Haberl N., Thalhammer S., Hauck S.M. (2017). Colloidal Stability and Surface Chemistry Are Key Factors for the Composition of the Protein Corona of Inorganic Gold Nanoparticles. Adv. Funct. Mater..

[29] Fuller M., Kӧper I. (2018). Polyelectrolyte-Coated Gold Nanoparticles: The Effect of Salt and Polyelectrolyte Concentration on Colloidal Stability. Polymers.

[30] Sadequa S., Nadia D., Sanda B.-F., Milena S., Nathalie C., Simion A., Hanna H., Marc Lamy de la C. (2015). Comparative toxicity evaluation of flower-shaped and spherical gold nanoparticles on human endothelial cells. Nanotechnology.

[31] Dykman L.A., Khlebtsov N.G. (2013). Uptake of Engineered Gold Nanoparticles into Mammalian Cells. Chem. Rev..

[32] Huang X., Jain P.K., El-Sayed I.H., A El-Sayed M. (2007). Gold nanoparticles: interesting optical properties and recent applications in cancer diagnostics and therapy. Nanomedicine.

[33] Li N., Zhao P., Astruc D. (2014). Anisotropic Gold Nanoparticles: Synthesis, Properties, Applications, and Toxicity. Angew. Chem. Int. Ed..

[34] Mahan M.M., Doiron A.L. (2018). Gold Nanoparticles as X-Ray, CT, and Multimodal Imaging Contrast Agents: Formulation, Targeting, and Methodology. J. Nanomater..

[35] Kaur P., Aliru M.L., Chadha A.S., Asea A., Krishnan S. (2016). Hyperthermia Using Nanoparticles—Promises and Pitfalls. Int. J. Hyperth..

