# Supplementary material for: Anisotropic Gold Nanoparticle-Cell Interactions Mediated by Collagen
Source: Materials (Basel). 2019 Apr 6;12(7):1131. doi: 10.3390/ma12071131 (PMC6480049; doi:10.3390/ma12071131)
Supplement: Supplementary file 1 [file materials-12-01131-s001.pdf]

materials

MDPI

Supplementary Information

Anisotropic Gold Nanoparticle-Cell Interactions Mediated by Collagen

Oana T. Marișca [1] and Nicolae Leopold [1,2,*]

1 Faculty of Physics, Babeș-Bolyai University, 400084 Cluj-Napoca, Romania; oanamarisca@gmail.com
2 IMOGEN Research Institute, County Clinical Emergency Hospital, 400012 Cluj-Napoca, Romania
* Correspondence: nicolae.leopold@phys.ubbcluj.ro; Tel.: +40-264-405300

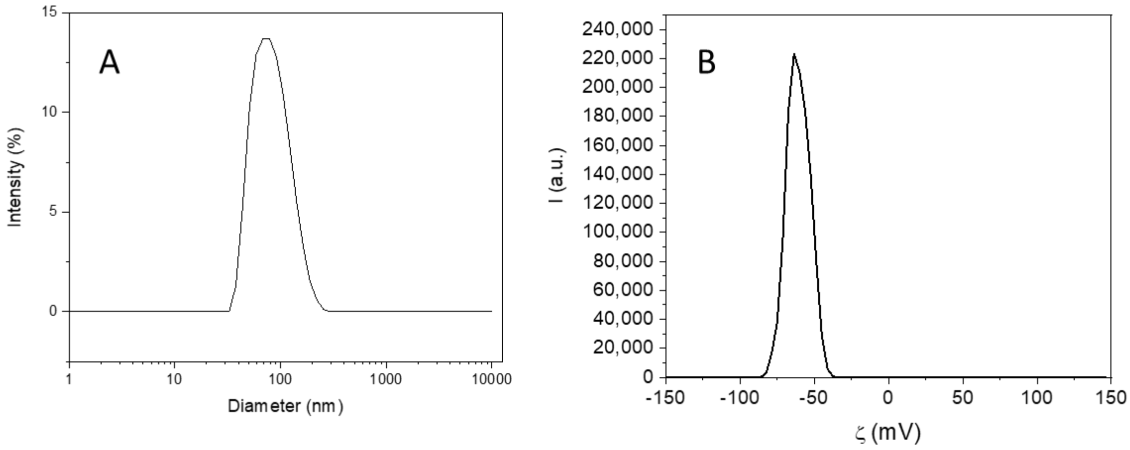

Figure S1. Size distribution of clg-AuNPs measured by dynamic light scattering (DLS) (A). Zeta potential distribution of clg-AuNPs (B).

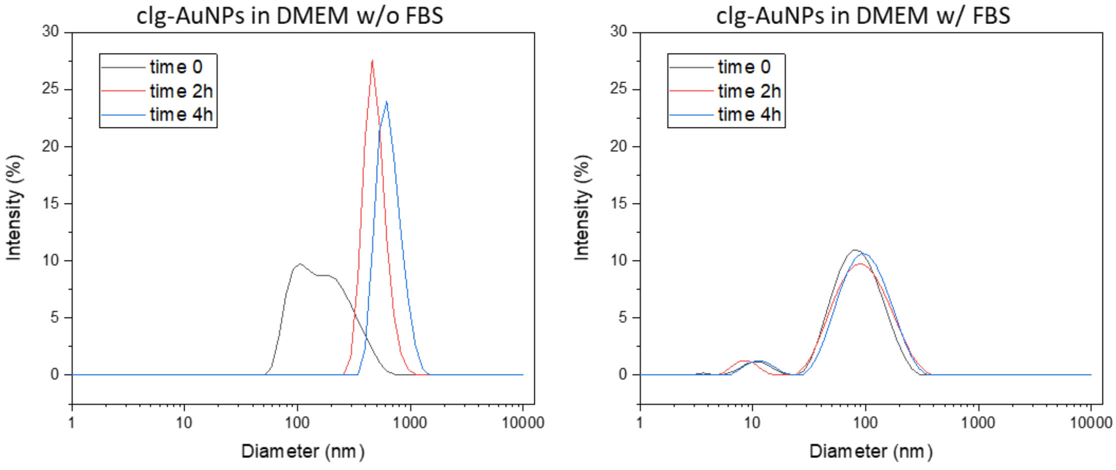

Figure S2. DLS measurements showing clg-AuNPs stability in cell culture medium without or with serum.

© 2019 by the authors. Licensee MDPI, Basel, Switzerland. This article is an open access article distributed under the terms and conditions of the Creative Commons Attribution (CC BY) license (http://creativecommons.org/licenses/by/4.0/).
